# STK35L1 Associates with Nuclear Actin and Regulates Cell Cycle and Migration of Endothelial Cells

**DOI:** 10.1371/journal.pone.0016249

**Published:** 2011-01-20

**Authors:** Pankaj Goyal, Antje Behring, Abhishek Kumar, Wolfgang Siess

**Affiliations:** 1 Institut für Prophylaxe und Epidemiologie der Kreislaufkrankheiten, Klinikum Innenstadt, Universität München, Munich, Germany; 2 Department of Biology, University of Padova, Padova, Italy; McMaster University, Canada

## Abstract

**Background:**

Migration and proliferation of vascular endothelial cells are essential for repair of injured endothelium and angiogenesis. Cyclins, cyclin-dependent kinases (CDKs), and cyclin-dependent kinase inhibitors play an important role in vascular tissue injury and wound healing. Previous studies suggest a link between the cell cycle and cell migration: cells present in the G_1_ phase have the highest potential to migrate. The molecular mechanism linking these two processes is not understood.

**Methodology/Principal Findings:**

In this study, we explored the function of STK35L1, a novel Ser/Thr kinase, localized in the nucleus and nucleolus of endothelial cells. Molecular biological analysis identified a bipartite nuclear localization signal, and nucleolar localization sequences in the N-terminal part of STK35L1. Nuclear actin was identified as a novel binding partner of STK35L1. A class III PDZ binding domains motif was identified in STK35L1 that mediated its interaction with actin. Depletion of STK35L1 by siRNA lead to an accelerated G_1_ to S phase transition after serum-stimulation of endothelial cells indicating an inhibitory role of the kinase in G_1_ to S phase progression. Cell cycle specific genes array analysis revealed that one gene was prominently downregulated (8.8 fold) in STK35L1 silenced cells: CDKN2A alpha transcript, which codes for p16^INK4a^ leading to G_1_ arrest by inhibition of CDK4/6. Moreover in endothelial cells seeded on Matrigel, STK35L1 expression was rapidly upregulated, and silencing of STK35L1 drastically inhibited endothelial sprouting that is required for angiogenesis. Furthermore, STK35L1 depletion profoundly impaired endothelial cell migration in two wound healing assays.

**Conclusion/Significance:**

The results indicate that by regulating CDKN2A and inhibiting G1- to S-phase transition STK35L1 may act as a central kinase linking the cell cycle and migration of endothelial cells. The interaction of STK35L1 with nuclear actin might be critical in the regulation of these fundamental endothelial functions.

## Introduction

Endothelial dysfunction underlies atherosclerosis and coronary heart disease [1], [2]. Migration and proliferation of vascular endothelial cells are important not only for repair of injured endothelium, but also for angiogenesis [3]. Cells in the endothelial monolayer are in a quiescent state residing in the G_o_ phase of the cell cycle. Injury of the endothelium leads to the local release of peptide growth factors (such as VEGF, TGF) and bioactive lipids (i.e. S1P) that stimulate endothelial cell migration and proliferation crucial for endothelial healing [4], [5]. Angiogenesis induced by hypoxic tissue conditions or by angiogenic stimuli, is a complex biological process involving the directional migration, proliferation, intercellular alignment and adhesion of endothelial cells [3]. Healing of the endothelium and angiogenesis require the activation of a genetic program which regulates endothelial cell proliferation and migration in a coordinated manner.

Cyclins, the cyclin-dependent kinases (CDKs), and the cyclin-dependent kinase inhibitors (CKIs) play an important role in vascular tissue injury, inflammation and wound repair [6], [7]. On stimulation by growth factors or after mechanical trauma, endothelial cells exit the quiescent state and progress through G_1_ and S phase of the cell cycle. G_1_ phase progression is regulated by the assembly and phosphorylation of CDK complexes. Two classes of endogenous inhibitors of the CKI are dominant in cardiovascular biology: the CIP/KIP family, which includes p21^Cip1^, p27^Kip1^, p57^Kip2^, and the INK4 family, which includes p15^Ink4b^, p16^Ink4a^, p18^Ink4c^, and p19^Ink4d^. p16^INK4a^ binds to cyclin/CDK complexes and causes cell cycle arrest in the G_1_ phase by inhibiting CDK4/6 mediated phosphorylation of Rb [8]. p16^INK4a^ and p15^INK4b^ are encoded by the alpha-transcript of CDKN2A and the CDKN2B gene, respectively. Recent genome-wide association scanning studies identified DNA sequence variants at chromosome 9p21 that increase the risk of coronary heart disease, myocardial infarction and, independently, type 2 diabetes [9], [10]. Interestingly, the genomic region of interest was found to be adjacent to the genes CDKN2A and CDKN2B. The mechanism by which these genes might influence coronary heart disease and type 2 diabetes is unknown.

Previous studies of vascular cells show that there is a link between cell cycle progression and migration [11], [12], [13]. The maximal potential of a cell to migrate lies in the mid-late G1 phase, whereas cells in the late S or G_2_/M phase have a lower or no ability to move [14], [15]. p27^Kip1^ has been shown to regulate G_1_-S phase cell cycle progression and cell migration of endothelial and smooth muscle cells [13], [16]. Endothelial cell migration requires dynamic changes of the actin cytoskeleton which is regulated by small GTPases and various protein kinases [17]. The molecular mechanisms linking in endothelial cells cell cycle progression and migration are not known.

STK35L1 is a member of the class of serine/threonine protein kinases; it is mainly localized in the nucleolus and nucleus [18]. Recently we identified the full length coding sequence of the *STK35L1* gene which codes for a protein of 534 amino acids [18]. The biological function of STK35L1 is not known. *STK35L1* gene expression was found to be upregulated in colorectal cancer [19], and was altered in a rodent model of Parkinson disease [20]. A kinome-wide RNAi screen revealed that STK35L1 silencing was among top five hits leading to reduce infection of hepatocytes by *Plasmodium berghei* sporozoites [21]. These studies suggest that STK35L1 may play a role in various human diseases.

In the present study we set out to explore the function of STK35L1 in endothelial cells. We show that STK35L1 regulates the expression of CDKN2A alpha-transcript and inhibits the G1- to S-phase transition of the cell cycle, and that STK35L1 is essential for endothelial cell migration and angiogenesis. STK35L1 might be part of a program underlying the integrated regulation of the cell cycle and cell migration.

## Results

### The N-terminal region of STK35L1 has functional nuclear and nucleolar localization signals

**Figure 1:**
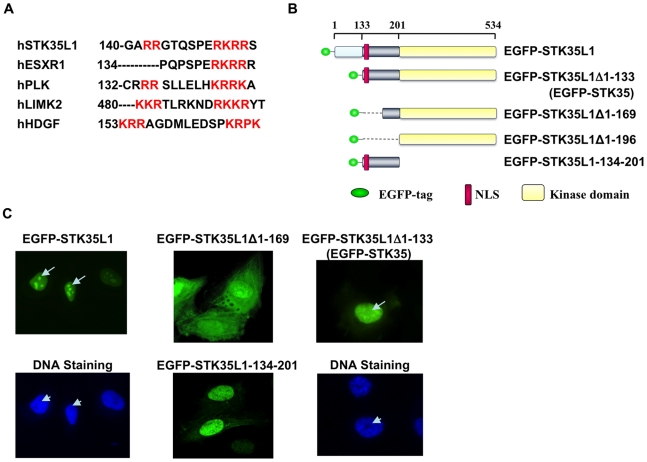
Nuclear and nucleolar localization signals in STK35L1. A) Prediction of nuclear localization signals in STK35L1. NLS was aligned with the known monopartite NLS of ESXR1 and bipartite NLSs of Plk1[22], LIMK2 [22] and HDGF [49]. Basic amino acids are shown in red colors. B) Schematic representation of different EGFP-STK35L1 deletion mutants. The upper axis with coordinates represents the position of amino acids. The predicted bipartite NLS (aa 142–153) is shown as red box. C) Subcellular distribution of different EGFP-STK35L1 deletion mutants. EGFP-STK35L1 was mainly localized in the nucleus and nucleolus (Top left panel; white arrow). The nucleolus appears as a dark spot excluded by the DNA staining with Hoechst-dye (lower left panel, white arrow head). The expression of the various EGFP-STK35L1 deletion mutants in endothelial cells is shown (see text for details).

We have previously shown that STK35L1 is mainly localized in the nucleolus and the nucleus [18]. To predict a functional nuclear localization signal (NLS) in STK35L1, the basic amino acid-rich motifs of STK35L1 were analyzed by manual comparison with known NLSs of different proteins. A potential bipartite NLS was identified within the N-terminal motif (amino acids (aa) 142–153) of the protein. Unlike the classical bipartite NLS consisting of a defined spacer of 8–10 non-basic amino acids, the identified potential NLS of STK35L1 has a 6-amino acids short spacer sequence similar to LIMK2 (Figure 1A) [22]. The identified NLS is highly conserved among mammals (Figure S1). Conservation of the identified bipartite NLS sequence underlies its functional importance.

To analyze whether the predicted NLS is functional, we prepared various EGFP-tagged deletion constructs of STK35L1 (Figure 1B). As compared with EGFP-STK35L1 that predominantly localized in the nucleus and nucleolus, the constructs deleted of the N-terminal 169 aa (EGFP-STK35L1Δ1-169) containing mainly the kinase domain, was distributed throughout the cytoplasm and the nucleus (Figure 1C). The construct containing the predicted NLS (EGFP-STK35L1-134-201) mainly accumulated in the nucleus (Figure 1C). These data suggest that the motif aa 142–153 is the functional bipartite NLS of STK35L1.

Nucleolar localization signal (NoLS) are sequences rich in arginine and lysine. So far, no specific consensus sequences for nucleolar localization have been determined. We observed that several stretches of arginine and lysine amino acids are present in the N-terminal part of STK35L1 that are also highly conserved among mammals (Figure S1). These analyses suggest a putative NoLS in the N-terminal region of STK35L1. We found that the mutant EGFP-STK35L1Δ1-133 (aa 1–133 were deleted) was excluded from the nucleolus but mainly localized in the nucleus (Figure 1C). This mutant corresponds to the protein previously described as STK35 [18]. Our data indicate that the N-terminal aa 1–133 contains a functional nucleolar localization signal (NoLS) in STK35L1 that is absent in STK35.

### EGFP-STK35 interacts with nuclear actin

**Figure 2:**
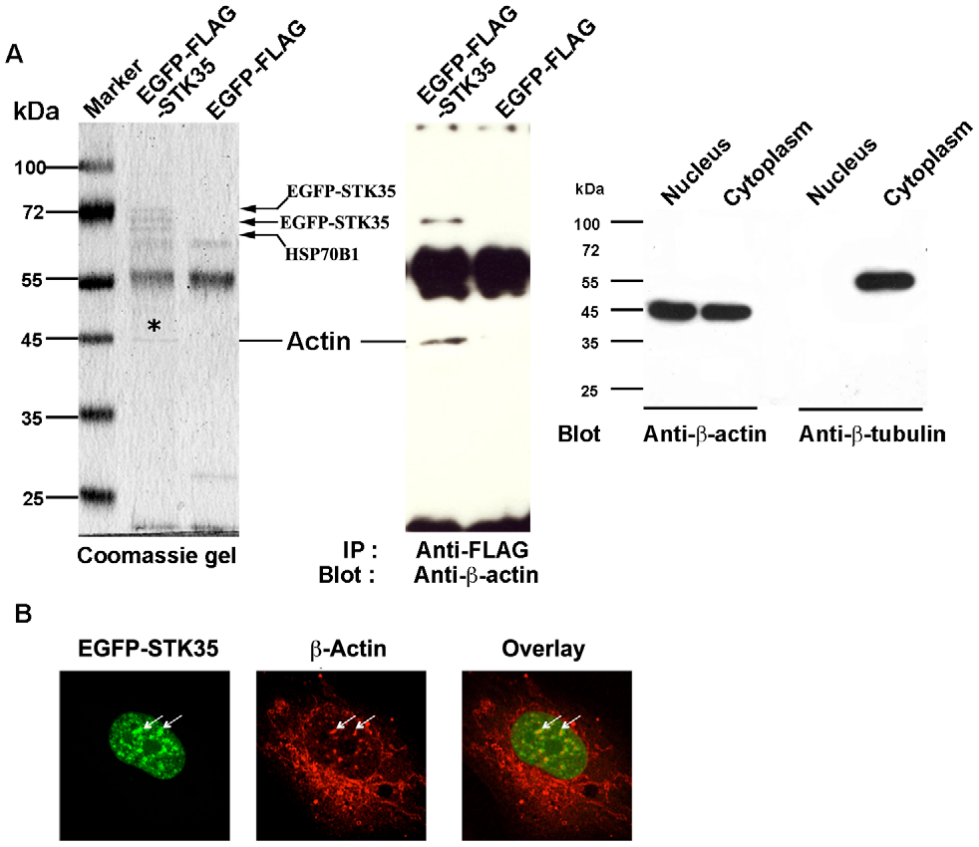
EGFP-STK35 intracts with nuclear actin. A) Nuclear actin coimmunoprecipitates with EGFP-FLAG-STK35. HEK293T cells were transfected with EGFP-FLAG-STK35 and EGFP-FLAG. The nuclei were isolated and EGFP-FLAG-tagged proteins were immunoprecipitated with anti-FLAG antibody. Bound proteins were resolved by SDS-PAGE and stained with Coomassie blue. The specific bands were cut, in-gel digested by trypsin and identified by MALDI-TOF and peptide mass fingerprinting. The protein band around 45 kDa (under “*”) was as β-actin (left panel). The other bands were identified as HSP70B1 and EGFP-FLAG-STK35. The same immunoprecipitated samples were blotted with monoclonal anti-actin antibody and the band of 45 kDa was confirmed as β-actin (middle panel). To check the purity of the nuclear preparation, 10 µl of 100 µl nuclear fraction and 10 µl of 4 ml cytoplasmic fraction were subjected to SDS-PAGE and blotted with an anti-actin and an anti-tubulin antibody. The apparent 50∶50 ratio of nuclear to cytoplasmic actin is due to the high amount of actin in the diluted cytoplasmic fraction. Tubulin (55 kDa) could be detected in the cytoplasmic but not in the nuclear fraction, demonstrating the nuclear fraction was free from cytoplasmic proteins (right panel). B) EGFP-STK35 and β-actin colocalized partially in the nucleus. Endothelial cells transfected with EGFP-STK35 (left panel, green) and stained for β-actin (middle panel, red) with anti-actin monoclonal antibody (clone C4) 8 h after transfection. The overlay (right panel) shows colocalization of EGFP-STK35 and β-actin. Arrows indicate some sites of colocalization.

STK35L1 is localized in the nucleus and nucleolus whereas STK35 is mainly localized in the nucleus [18]. To identify proteins which interact with STK35L1 in the nucleus, we immunoprecipitated EGFP-FLAG-STK35 protein from nuclear extracts of EGFP-FLAG-STK35-transfected HEK293 cells using an anti-FLAG antibody. We found four co-immunoprecipitated proteins which were identified by MALDI-MS analysis as EGFP-STK35 (2 bands), HSP70B1 and actin (Figure 2A, left panel). The co-immunoprecipitation of actin with EGFP-FLAG-STK35 was confirmed by western blotting using a specific anti-β-actin antibody (Figure 2A, middle panel). The purity of the nuclear preparation was checked by probing it with an anti-β-tubulin antibody that detected tubulin in the cytoplasmic fraction only indicating that the nuclear preparation was free from cytoplasmic proteins (Figure 2A, right panel). β-actin was present in both the nucleus and the cytoplasm. These data suggest that STK35L1 interacts with nuclear actin.

To investigate further a possible colocalization of STK35 with nuclear β-actin, endothelial cells were studied by fluorescence microscopy. In transfected endothelial cells, EGFP-STK35 (green) was concentrated in dot-like structures within the nucleus (Figure 2B left panel). In some of these nuclear structures, β-actin (red) was colocalized with EGFP-STK35 (Figure 2B right panel). Together these data suggest that STK35L1 interacts with actin in the nucleus of endothelial cells.

### A potential class III PDZ domain binding motif of STK35L1 is responsible for its association with actin

**Figure 3:**
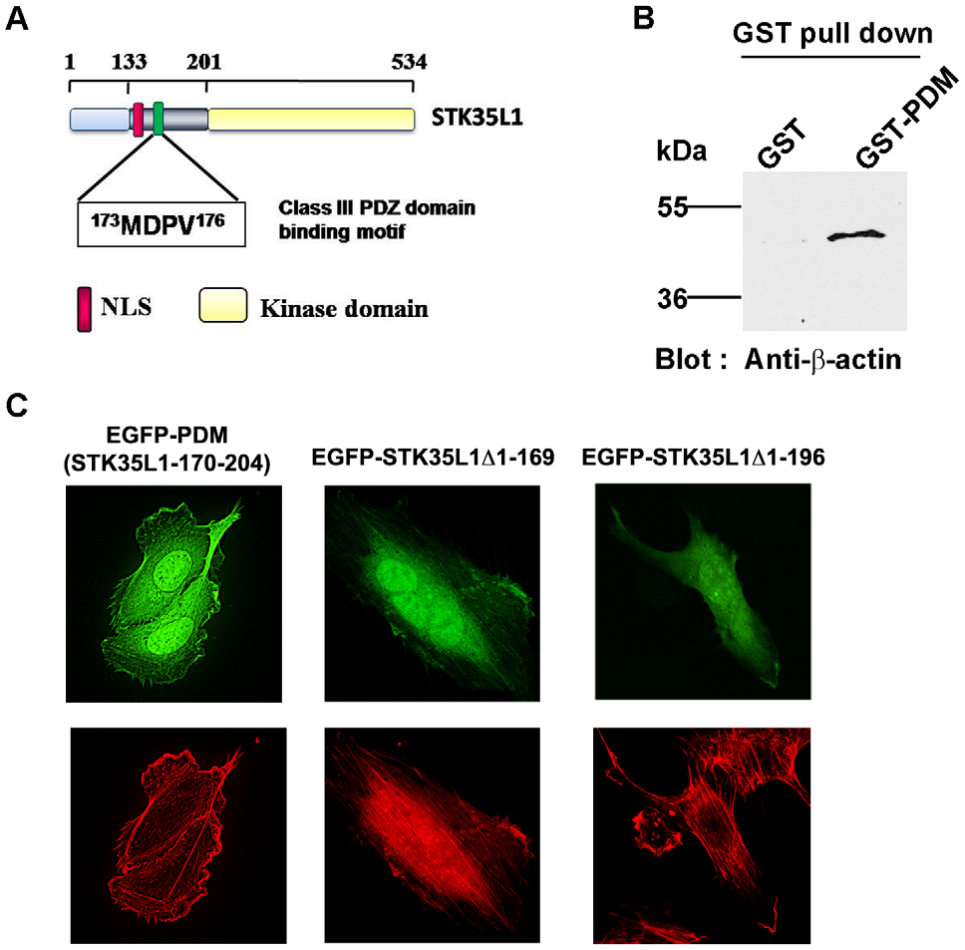
Identification of Putative class III PDZ-binding domains motif in STK35L1 that mediates its interaction with actin. A) Class III PDZ binding domains motif (PDZ-BM) was predicted by ELM software and its position within the STK35L1 sequence is indicated as green box; kinase domain: yellow box, NLS: red box. B) GST pull down assay of nuclear lysates from HeLa cells was preformed using recombinant GST or GST-PDM protein (GST coupled to 30 amino acids (aa position 170 to 204) of STK35L1 containing the potential PDZ-BM). Bound proteins were resolved by SDS-PAGE and immunoblotted using a monoclonal anti-β-actin antibody C) Subcellular distribution of different EGFP-STK35L1 deletion mutants in transfected endothelial cells. EGFP-PDM (aa 170–204, contains only the PDZ-BM of STK35L1) was localized to actin stress fibers. EGFP-STK35L1Δ1-169 mutant (lacking NoLS and NLS) was distributed throughout the nucleus and cytoplasm with a partial localization to fiber like structures of the cytoskeleton. EGFP-STK35L1Δ1-96 (lacking NoLS, NLS and PDZ-BM) was diffusely distributed throughout the cytoplasm and the nucleus but no green fiber like structures were visible.

To gain insight into protein-protein interaction domains of STK35L1, we used the Eukaryotic Linear Motif server (ELM) for investigating candidate short non-globular functional motifs within the STK35L1 protein [23]. Several protein-binding motifs were predicted that were mainly located in the first 200 amino acids of STK35L1 (Table S1). Among the motifs, an internal class III PDZ domain-binding motif (PDZ-BM) was predicted in the N-terminal part of STK35L1 (aa 173–176; Figure 3A). PDZ domains are found in many proteins, also in proteins, that are involved in the regulation of the actin cytoskeleton [24], [25]. To test whether the PDZ-BM is responsible for actin association, we performed GST pull down assay of nuclear lysates from HeLa and endothelial cells using a recombinant GST-tagged protein containing the potential PDZ-BM (GST-PDM). GST-PDM consists of GST coupled to 30 amino acids (aa position 170 to 204) of STK35L1 containing the potential PDZ-BM. Indeed, we found that the purified protein GST-PDM but not GST bound with nuclear actin (Figure 3B).

To further study, whether the PDZ-BM associates of STK35L1 with actin *in vivo*, we studied the localization of EGFP-PDM (STK35L1-170–204) in transfected endothelial cells. We found that EGFP-PDM, present both in the cytoplasm and the nucleus due to the small size of the protein and the lack of NLS and NoLS sequences, strongly associated with actin stress fibers (Figure 3C, left panel). EGFP-PDM was not only localized on stress fibers, it was also enriched in membrane ruffles of migrating endothelial cells (Movie S1).

To study, whether the PDZ-BM is responsible for the association of STK35L1 with actin, we investigated the subcellular distribution of different EGFP-tagged deletion mutants of EGFP-STK35L1 in transfected endothelial cells. The deletion mutant containing the PDZ-BM but lacking the NoLS and NLS (EGFP-STK35L1Δ1-169) resulted in a more cytoplasmic distribution of the fusion protein than EGFP-STK35L1 with a partial localization on fiber-like structures of the cytoskeleton (Figure 3C, middle panel). The deletion mutant that lacked the PDZ-BM in addition to NoLS and NLS (EGFP-STK35L1Δ1-196) was diffusely distributed throughout the cytoplasm and the nucleus (Figure 3C, right panel). In none of the transfected cells a fiber-like structure could be observed.

Together, we suggest that a class III PDZ domain binding motif is responsible for the association of STK35L1 with actin.

### SiRNA-mediated depletion of STK35L1 accelerates G1-S phase progression of the endothelial cell cycle

**Figure 4:**
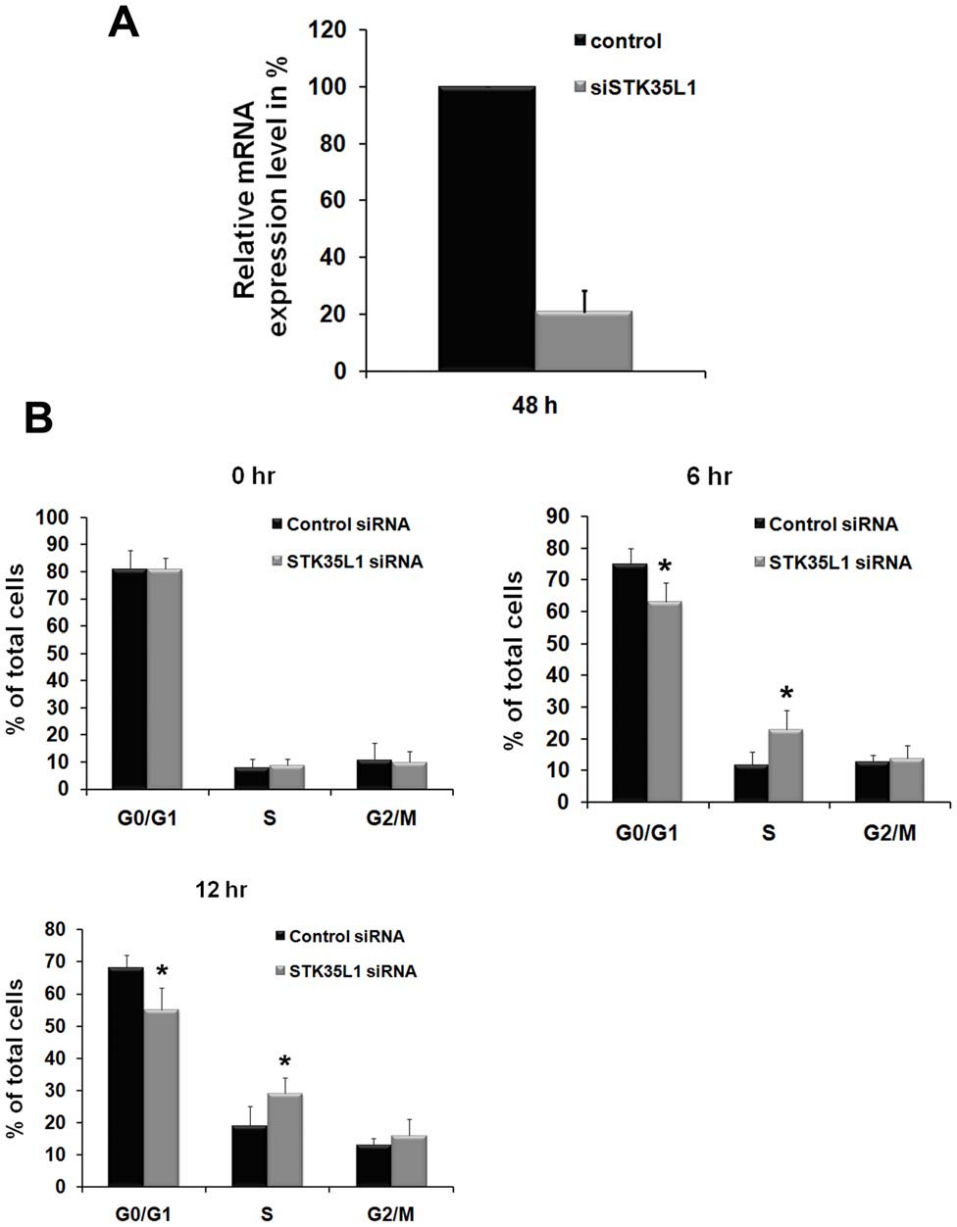
G_1_ to S-phase progression is accelerated in STK35L1-silenced endothelial cells. A) STK35L1 expression level of STK35L1 silenced cells and control cells. B) Cell cycle distribution of control siRNA- and STK35L1 siRNA treated endothelial cells. Cells arrested in G_o_/G_1_ phase were stimulated by serum for various times. Values are mean ± S.E. of three independent experiments. Asterisk “*” indicates level of significance p≤0. 05.

To analyze the role of nucleolar STK35L1 in the cell cycle, we used siRNA to silence the STK35L1 gene. We designed siRNA directed against three different regions of the STK35L1 gene [18] which together down-regulated STK35L1 by 80–90% at the level of transcription (Figure 4A) and at the level of protein [18]. siRNA- or nonspecific siRNA-transfected cells were synchronized in G_0_/G_1_ phase by serum depletion, and thereafter the cells were released into cell cycle progression by adding 10% serum. In synchronized cells, over 80% of control and STK35L1-silenced cells accumulated in the G_0_/G_1_ phase. Six hours after release, the number of control cells in the G_1_/G_0_ phase was decreased by 6%, and 12% of control cells were present in the S phase (Figure 4B). In STK35L1-silenced cells, the G_1_-S phase transition was accelerated: the number of cells in the G_1_/G_0_ phase was decreased by 18% (p<0.05), and the number of cells in the S phase was increased almost two fold (23%; Figure 4B, 6 hr). Twelve hours after release, 68% (±4%) of control cells were in the G_1_/G_0_ phase and 19% (±6%) had entered S phase. After STK35L1 silencing only 55% cells remained in G_1_/G_0_ phase, and 29% of cells had entered the S phase (Figure 4B).

These data indicate that knockdown of the STK35L1 gene leads to an accelerated G_1_ to S phase transition. STK35L1 might therefore function as a negative regulator of the cell cycle progression from G_1_ to S phase.

### STK35L1 regulates the expression of G_1_/S phase specific genes

To determine the influence of STK35L1 on genes involved in cell cycle regulation, a real-time RT-PCR-based commercial gene array was used. This gene array measures the expression of 84 genes that include positive and negative regulation of the cell cycle, phase transitions, checkpoints, and DNA replication (Table S2). Endothelial cells were transfected with STK35L1 siRNA or control siRNA, followed by cell synchronization in G_0_/G_1_ phase and then released by serum stimulation.

Since we found in STK35L1-silenced cells an acceleration of the G_1_/S- phase transition, we expected that by STK35L1-silencing the expression of those genes might be altered, which function during G1/S phase transition. Indeed, the most prominently downregulated gene (8.8 fold) was p16^INK4a^ (Table 1). This protein is encoded by the alpha-transcript of *CDKN2A* gene [8], inhibits the cell cycle and is responsible for arresting cells in the G_1_-phase. We were unable to detect p16^INK4a^, which is expressed probably at a very low level in HUVEC by immunoblotting using commercial antibodies in HUVEC. Also another study was unable to detect p16^INK4a^ in HUVEC under normal growth condition [26].

**Table 1:** mRNA expression of selected cell cycle specific genes in STK35L1-silenced cells.

Reference Sequence	Description	Gene Symbol	Fold Regulation	p-value
NM_004701	Cyclin B2	CCNB2	−3.605	0.060
NM_001786	Cell division cycle 2	CDC2	−2.9966	0.030*
NM_001255	Cell division cycle 20 homolog (S. cerevisiae)	CDC20	−2.9282	0.055
NM_001259	Cyclin-dependent kinase 6	CDK6	−2.7321	0.005*
NM_000077	Cyclin-dependent kinase inhibitor 2A (p16^INK4A^)	CDKN2A	−8.8766	0.050*
NM_005192	Cyclin-dependent kinase inhibitor 3	CDKN3	−3.4422	0.060
NM_004399	DEAD/H (Asp-Glu-Ala-Asp/His) box polypeptide 11	DDX11	−4.7568	0.014*
NM_001924	Growth arrest and DNA-damage-inducible, alpha	GADD45A	−4.6482	0.030*
NM_016426	G-2 and S-phase expressed 1	GTSE1	−2.9966	0.029*
NM_002417	Antigen identified by monoclonal antibody Ki-67	MKI67	−2.7959	0.015*
NM_002875	RAD51 homolog	RAD51	−2.8613	0.022*
NM_003885	Cyclin-dependent kinase 5, regulatory subunit 1 (p35)	CDK5R1	2.09	0.078

Mean fold change of mRNA expression of selected cell cycle specific genes in STK35L1-silenced cells vs. control cells.

*Statistically significant (P value of ≤0.05).

Two other genes were significantly down-regulated in STK35L1-silenced cells: GADD45A, a protein involved in DNA repair as well as in G_1_ cell cycle arrest [27], and DDX11, which encodes a nucleolar helicase (Table 1). No gene was significantly upregulated in STK35L1 silenced cells. These data suggest that STK35L1 might function as a negative regulator for the cell cycle progression by affecting the expression levels of genes, which inhibit the G_1_- to S-phase transition, such as p16^INK4a^ and GADD45A.

### STK35L1 is upregulated during angiogenesis

**Figure 5:**
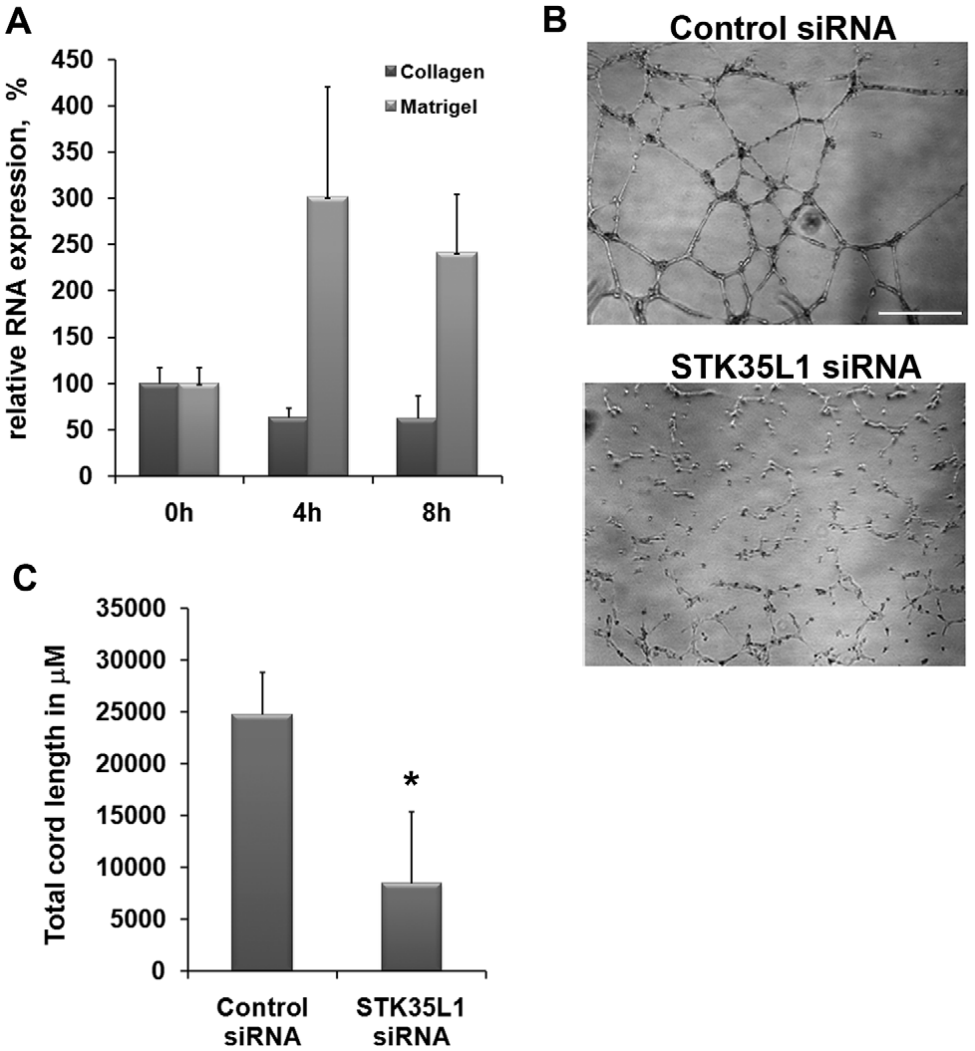
STK35L1 regulates endothelial morphogenesis. A) STK35L1 expression in endothelial cells cultivated in the presence of VEGF on collagen or on Matrigel. The expression level of STK35L1 transcripts at the indicated time points was analyzed by RT-PCR. The relative expression level compared to time 0 hr is shown. B) and C) STK35L1 regulates the formation of cord-like structures on Matrigel. Control siRNA and STK35L1-siRNA transfected cells were grown on Matrigel for 6 h. B) phase contrast micrograph, Scale bar 500 µm. (C) Bar diagram, values are mean ± S.E. of three independent experiments; * p<0.05.

Endothelial cells cultivated on a basal membrane-like matrix such as Matrigel, undergo a rapid morphogenesis: they migrate on the matrix, form cell–cell contacts and a network of cords, but they do not proliferate. In contrast, cells cultivated on collagen-coated surfaces undergo mainly proliferation [28]. We reasoned that STK35L1 expression might inhibit endothelial cell proliferation on basal membrane-like matrix, and perhaps increase the migration potential of the cells by keeping them in the G_1_-phase. Therefore, we compared first the effect of the two different matrices (Matrigel and collagen) on STK35L1 expression of endothelial cells. Indeed we found that the expression of STK35L1 transcripts was increased threefold within 4 hours only in the cells growing on basal membrane-like matrix (Figure 5A).

### Knockdown of STK35L1 inhibits the formation of endothelial sprouting

Next, we analyzed whether silencing of STK35L1 affects the morphogenesis of endothelial cells on Matrigel. Endothelial cells transfected with control siRNA formed six hours after plating a network of cord-like structures on basal membrane-like matrix (Matrigel) similar to non-transfected cells (Figure 5B). In contrast, in STK35L1-silenced cells endothelial sprouting was drastically inhibited (by 70%; Figure 5B, 5C). The silenced cells also showed considerably fewer nodes with branching outlets (Figure 5B lower panel). These data indicate that STK35L1 regulates the morphogenetic process required for angiogenesis.

### STK35L1 silencing inhibits endothelial cell migration

**Figure 6:**
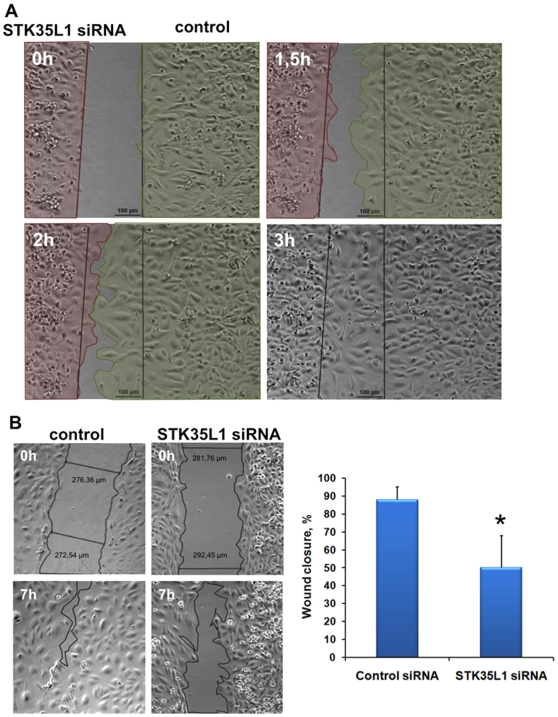
STK35L1 is required for endothelial cell migration. A) Migration assay using the IBIDI insert. Endothelial cells transfected with STK35L1 siRNA (red shaded part) or control siRNA (green shaded part) were seeded into different reservoirs of an IBIDI insert. After 8 hours the insert was removed and the closure of the gap was observed by video microscopy. The pictures from one experiment representative of three are shown, Scale bar 100 µm. B) Migration after mechanical injury. Left panel, representative micrographs, cells treated with control siRNA, or cells treated with STK35L1 siRNA are shown immediately 0 hr or 7 hr after mechanical injury; width of the wound is shown in µm. Right panel, Bar diagram, wound closure was quantified as described in the method section. Values are mean ± S.E. of three independent experiments; ‘*’, p<0.05.

In order to find out, which step of morphogenesis is affected in STK35L1-silenced cells, we analyzed the migration of STK35L1-silenced endothelial cells by using two different wound-healing assays. In the assay using the IBIDI Culture-Insert where no cell damage occurs, the migration of STK35L1-silenced cells was drastically inhibited: the cells were not able to move in the direction of the wound (Movie S2, Figure 6A). In STK35L1-silenced cells, we could not observe the stable lamellipodia formation in the direction of migration (like in control), but we found transient lamellipodia formation in all the directions (Movie S2). Also after mechanical injury of confluent endothelium, where cell damage occurs, the migration of STK35L1-silenced cells was inhibited (Figure 6B, 4C). These results show that STK35L1 is crucial for endothelial cell migration.

## Discussion

The present study demonstrates that the localization of the novel kinase STK35L1 in the nucleus and nucleolus is regulated by a specific bipartite NLS, and a NoLS both present in the N-terminal part of the protein. We found an interaction of STK35L1 with nuclear actin that is mediated through a potential PDZ-BM motif present in the N-terminal part of STK35L1. Furthermore, STK35L1 regulates the expression of CDKN2A and inhibits the G1-to S-phase transition of the cell cycle, and STK35L1 is essential for endothelial cell migration and cord-like structure formation on Matrigel.

The N-terminal part of STK35L1 is highly basic and rich in arginine and lysine and contains the bipartite NLS and NoLS. The identified bipartite NLS sequence was conserved among mammals, and we showed that it was functional in endothelial cells. Our study further shows that the bipartite NLS (aa 142–153) did not overlap with the NoLS sequences (aa 1–133) of STK35L1. Nucleolar localizing proteins are rich in arginine and lysine and often overlap with the NLS, suggesting a complex regulation of nucleolar localization [29]. Other studies have identified small NoLS sequence motifs ranging from 7 to 30 residues have been identified that can be sufficient to target a protein to the nucleolus [22], [30]. We found that NoLS of STK35L1 had no well-defined small sequence motif.

In terms of functions, we show that STK35L1 is a negative regulator of the G_1_ to S phase progression of the endothelial cell cycle by increasing directly or indirectly the expression of the cell cycle inhibitor CDKN2A. Previously, high expression of CDKN2A was correlated with a low proliferation of colorectal carcinomas at the invasion front [31]. Moreover, enhanced levels of STK35L1 transcripts have been reported in tissues obtained from colorectal cancer patients [19]. Interestingly, by using Phosphoproteomics of the Kinome across the Cell Cycle, STK35 (N-terminal 133 aa truncated-form of STK35L1) was found to be phosphorylated during the cell cycle progression in HeLa S3 cells. This study identified Thr-12 in STK35 as phosphorylated amino acid, which corresponds to Thr-145 in STK35L1 [32]. Notably, Thr-145 is within the bipartite NLS sequence (STK35L1 aa 142–153) identified in the present study. Many proteins such as LIMK2, Ca^2+^/calmodulin-dependent protein kinase II, and cyclin B1 are phosphorylated near or within their NLS thereby affecting their nucleocytoplasmic shuttling [22], [33], [34]. These results are interesting and lead us to suggest that phosphorylation of STK35L1 within its NLS during cell cycle progression might regulate its localization in the nucleus and/or nucleolus.

Our study shows that STK35L1 is upstream of CDKN2A. STK35L1 regulation of the CDNKN2A alpha-transcript (p16^INK4a^) could be a new signaling pathway regulating G_1_ to S phase progression of the cell cycle. It has been shown that nuclear β-catenin stimulates the expression of p16^INK4a^ in various tumor cell lines but the mechanism of regulation is not well understood [31], [35]. Interestingly, GSK3β-dependent phosphorylation regulates proteasomal degradation of β-catenin [36] and STK35 is a potential candidate for GSK3β-regulated proteasomal degradation [37]. Therefore, there are now two possibilities for regulation of CDKN2A (p16^INK4a^): either a novel GSK3β/STK35L1 pathway regulates independently of GSK3β/β-catenin CDKN2A, or STK35L1 is part of the GSK3β/β-catenin pathway regulating CDKN2A. These possibilities should be explored further as CDKN2A expression is important in various human diseases.

We found a crucial role of STK35L1 in endothelial cell migration as measured by two different cell migration assays. The question arises, how nuclear/nucleolar STK35L1 can regulate cell migration? Previous studies showed that G_1_ to S phase progression and cell migration are coordinated processes in different cell type: cells in mid-late G_1_ phase have the greatest ability to migrate, whereas cells in G_0_, S or G_2_/M phase have a lower or no ability to move [14], [15]. Therefore, it is possible that STK35L1 might promote endothelial cell migration by keeping cells in the G_1_-phase. Many cell cycle proteins have been reported to be involved in the regulation of cell migration [11], [13], [38], [39]. In endothelial cells and vascular smooth muscle cells, the CDK inhibitor p27^Kip1^ blocks cell migration [11], [13], however in other studies p27^Kip1^ has been reported to promote migration by interacting with the G_1_/S-phase specific cyclin D1 [38]. The subcellular distribution of p27^Kip1^ was found to be important in its promigratory function: cytoplasmic but not nuclear p27^Kip1^ promoted cellular migration [39]. In our study, we could not find an altered subcellular distribution of STK35L1 during endothelial cell migration suggesting that the role of STK35L1 in regulating migration is restricted to its nuclear/nucleolar localization.

Endothelial cell migration is required for angiogenesis. Endothelial cells on Matrigel migrate, polarize and proceed to form cord-like structures, but they do not proliferate [28]. Indeed we found that STK35L1 is upregulated in endothelial cells growing on Matrigel for 4 hours but not on collagen, and that STK35L1 was crucial for endothelial sprouting. We suggest that STK35L1 regulates this process by keeping endothelial cells in the G_1_ phase and thereby promoting cell migration.

We identified nuclear actin as interacting protein of STK35L1. The interaction of STK35L1 with nuclear actin might be important for the regulation of both the cell cycle and the migration of endothelial cells [40], [41], [42]. It is now well established that actin is present in various nuclear compartments such as the nucleolus. Nuclear actin forms structures different of cytosolic actin structures such as stress fibers [41]. Nuclear actin plays a role in gene transcription by regulation of transcription factors or as a component of chromatin remodeling complexes and RNP particles, and it is closely associated with all RNA polymerases [42]. For example, nuclear actin regulates the Serum response factor (SRF) by interacting with MAL, a Myocardin family transcription factor. SRF activity is a key event during cellular differentiation of many processes and transcriptionally controls many genes such as actin isoforms (*Actb*, *Actg*, *Acta2*) and actin-binding proteins (ABPs; e.g., *Gsn*) [43], [44]. Recently, It has been shows that SRF's play a crucial function during cell migration. In neuronal cells, cell migration is not only depends on cytoplasmic actin dynamics but also on the nuclear actin dependent functions such as gene transcription [45]. The nuclear actin/STK35L1 complex might regulate gene transcription of specific cell cycle proteins such as CDKN2A and genes involved in cell migration. [45]

Based on our results, it is not clear whether STK35L1 interacts directly or indirectly with actin. We identified a putative class III PDZ domain binding motif in the N-terminal region of STK35L1 that bound to actin. This suggests that STK35L1 may be a ligand for a PDZ domain containing protein. Since actin does not contain a PDZ domain, it is unlikely that STK35L1 through its PDZ domain-binding motif interacts directly with actin. The interaction should then be mediated by PDZ domain containing actin-binding proteins. Various PDZ containing proteins such as PDZ-LIM family proteins (CLP36) are known to interact with actin indirectly through binding to actin binding proteins [25], [46]. For example, the binding of CLP-36 to stress fibers is mediated by its binding to actinin [24]. It is not known whether nuclear actin also interacts with a PDZ domain containing protein.

Together the present study unravels an important new player in the orchestrated regulation of cell proliferation and migration of endothelial cells. STK35L1 by inhibiting the endothelial cell cycle and being essential for migration is important in regulating vascular healing and angiogenesis. The interaction of STK35L1 with nuclear actin might be critical in the regulation of these cellular processes.

## Materials and Methods

### Materials

Oligonucleotides and siRNAs were synthesized by MWG Biotech AG (Ebersberg, Germany). Anti-β-actin was purchased from Chemicon, Germany. Anti-p16^INK4A^ antibody was purchased from Cell Signaling Technology and anti-β-tubulin antibody from Abcam, Germany. FLAG-M2 gel slurry was purchased from Sigma, Germany. Complete mini protease inhibitors tablets were purchased from Roche Diagnostics, Germany. Glutathione beads and GSTrap FF columns were purchased from GE Healthcare, Lifesciences, Germany.

### Construction of the Expression Plasmids and site-directed mutagenesis

The full-length coding sequence of STK35L1 was cloned into pEGFP-C1 vector as described previously [18]. The deletion mutants of pEGFP-STK35L1 were generated by Quick-Change II site-directed mutagenesis kit (Stratagene) as per manufacturer's instructions. To prepare GST-PDM construct, the PDZ binding motif (PDZ-BM) containing region (aa position 170 to 204) of STK35L1 was amplified by PCR. The PCR-amplified product was cloned into EcoRI and XhoI sites of into pGEX-5X-1 plasmid (GE healthcare, Lifesciences) to obtain PDZ-BM containing protein fragment fused with GST.

### Cell Culture and Transfection

HUVECs (Human Umbilical Vein Endothelial cells) were obtained and cultured as described previously [47]. Briefly, endothelial cells (HUVECs) harvested from umbilical cords were plated onto collagen-coated plastic culture flasks, and were cultured at 5% CO_2_, and 37°C in complete endothelial growth medium (endothelial cell basal medium with supplements; Promo Cell, Germany). In all experiments, HUVECs were transfected with 5 µg DNA per 1×10^6^ cells using the HUVEC nucleofactor kit from Amaxa GmbH.

### Isolation of cell nuclei

Nuclei of EGFP-FLAG-STK35 or EGFP-FLAG-transfected or non-transfected endothelial and HEK 293T [18] and HeLa cells (DSMZ - Deutsche Sammlung von Mikroorganismen und Zellkulturen GmbH) were isolated using nuclei Isolation Kit: Nuclei EZ prep (Sigma) as per manufacturer's instructions. Briefly, the cells grown in tissue culture Ø 10 cm dish, washed with ice cold PBS twice and then 4 ml of ice cold Nuclei EZ lysis buffer was added to each dish. The cells were harvested and lysed by thoroughly scraping and then transferred to a 15 ml centrifuge tube, The nuclei were collected by centrifugation at 500×g for 5 minutes at 4°C and the nuclei pellet was washed by resuspending in cold Nuclei EZ lysis buffer. The washed nuclei were collected by centrifugation at 500×g for 5 minutes at 4°C and use for immunoprecipitation or GST pull down assay.

### Immunoprecipitation of EGFP-FLAG-STK35 from nuclear lysates

Immunoprecipitation from nuclear lysate was performed using the Nuclear CO-IP-Kit (Active motif) according to the manufacturers' recommendations. In brief, the nuclei, EGFP-FLAG-STK35-transfected cells were resuspended in 50 µl complete digestion buffer (CDB). Enzymatic shearing cocktail (0.25 µl) was added in the nuclei suspension, incubated for 90 minutes at 4°C. Subsequently, the reaction was stopped by addition of 1 µl EDTA (0.5 M). After gentle vortexing the tube was incubated 5 minutes on ice. The nuclear debris was removed by centrifugation for 10 minutes at 14,000×g 4°C. The supernatant, containing nuclear proteins, was diluted in 500 µl IP-incubation buffer, and 30 µl of anti-FLAG-M2 gel-slurry (Sigma; 3× washed with 5 volumes of IP-incubation buffer) was added to the solution and incubated overnight at 4°C. The next day, the suspension was centrifuged 30 sec 4000×g at 4°C The beads were washed 6 times with IP-wash buffer and finally resuspended in 60 µl 1× Laemmli-buffer and boiled for 5 minutes at 95°C and subjected to SDS-PAGE and western blotting.

### Western blotting

Western blotting was done as described previously [47] using as primary antibodies anti-β-actin (1∶100 000), anti-p16^INK4A^ (dilution 1∶500 to 1∶200) and anti-β-tubulin antibody (1∶500 dilution).

### Mass spectroscopy

The coimmunoprecipitated and coomassie-stained protein bands were excised from the SDS-PAGE gel and sent to the Zentrallabor für Proteinanalytik (Ludwig-Maximilians-Universität Munich, Germany) for protein identification by MALDI TOF-MS analysis. There, the proteins were in gel digested to peptides by the endoproteinase trypsin. Peptides were eluted and directly spotted on a MALDI sample plate. MALDI-TOF measurements were performed, and the resulting spectra were then analyzed via Mascot software (Matrix Science, London, United Kingdom) using the NCBI Protein Databank.

### Expression and purification of recombinant GST and GST-PDM

GST and GST-PDM plasmids were overexpressed in BL21 (DE3) pLysS (Stratagene) at 37°C after the addition of 0.5 mM isopropyl β-d-thiogalactoside at an *A*_600_ of ∼0.6 for three hours. Bacteria were harvested by centrifugation and resuspended in 10 ml of ice cold PBS buffer containing lysozyme (1 µg/ml) and complete mini protease inhibitors (Roche Diagnostics) and incubated on ice for 30 minutes. The bacterial cells were lysed by sonication and then Triton X-100 (1%) was added. Cell debris was removed by centrifugation at 60000×g and the supernatant was loaded on GSTrap FF column (GE Healthcare, lifesciences) pre-equibilirated with PBS. The column was then washed with PBS and bound GST-tagged protein was eluted with elution buffer (50 mM Tris base, 10 mM Glutathione; pH 8.0). The eluted fractions were pooled, and concentrated and desalted using Centricon® Plus-20 (Millipore).

### GST pull down assay

The nuclei from three culture flasks (75 cm^2^) of confluent endothelial cells were isolated, and nuclear protein extracts were prepared as described above. Nuclear protein extract (1 ml) was aliquoted equally in two microcentrifuge tubes. Purified GST or GST-PDM protein was added to each tube and incubated overnight at 4°C. Glutathione-Sepharose beads (GE-healthcare, Lifesciences; 50 µl; 3 times washed with 5 volumes of IP-incubation buffer) were added and samples were incubated for one hour at 4°C. The beads were pelleted by centrifugation, washed 6 times with IP-wash buffer and finally resuspended in 60 µl 1× Laemmli-buffer and boiled for 5 minutes at 95°C and subjected to SDS-PAGE and western blotting.

### Immunofluorescent staining and fluorescence microscopy

After 8–10 hours of transfection, cells were washed and fixed with 3.7% formaldehyde in PBS for 10 minutes at 4°C and then washed briefly 2 times with PBS. For permeabilization, the cells were incubated in 0.2% TritonX100/PBS for 10 minutes at room temperature followed by 3 times washing with PBS. For immuno-staining of nuclear actin, the fixed and permeabilized cells were incubated with blocking solution (2% fatty acid free BSA in PBS) for 30 minutes at room temperature, briefly rinsed with PBS and then incubated with anti-actin monoclonal antibody (clone 4, 1∶100 dilution) for one hour in humidified chamber. Cells were washed three times with PBS and incubated with Alexa Fluor®568 goat anti-mouse secondary antibody (1∶200 dilution) for 45 minutes at room temperature and then washed 3 times. For DNA staining, cells were incubated with Hoechst 33258 dye (1 µg/ml) for 10 minutes. Cells were observed with a Nikon TE2000E-PFS fluorescence microscope.

### STK35L1-silencing

STK35L1 specific siRNAs were designed directed against three different regions of STK35L1 gene[18]. For silencing, HUVECs were grown to 90% confluence in 6-well plates in complete endothelial cell growth medium. Before 24 hours of transfection, the cell growth medium was changed to OptiMEM medium containing 0.5% FCS without antibiotics. HUVEC were transfected with a pool of three siRNA using Oligofectamine™ (Invitrogen, Germany) for 48 hours according to manufacturer's protocol.

### Endothelial cell viability and cell proliferation assay

To measure endothelial cell viability, cells were centrifuged, and resuspended in PBS buffer after trypsinization. Cells were mixed with trypan blue (0.4%; Sigma) in a 1∶1 ratio and were incubated for 3minutes at room temperature. Cell viability was calculated by counting unstained (viable cells) and stained cells using a hemocytometer.

For endothelial cell proliferation assay, cells were grown in 24 well plates in complete endothelial growth medium. AlamarBlue® (Invitrogen) [48] reagent was added (1/10^th^ of volume of growth medium in each well) at different time points (24 hrs, 48 hours and 72 hours) and incubated for 4 hours at 37°C. The absorbance was measured at 570 nm and at 690 nm as reference wavelength (normalized to the 690 nm value) using Mithras LB 940 Multimode Reader. Cell proliferation is directly correlated to the absorbance value of developed color. The absorbance of control siRNA treated cells was considered as 100% and the proliferation was calculated as % of control (Figure S2).

### RT-PCR

To measure the STK35L1 expression in silenced-cells, or in other experiments, the cells, grown on collagen or on Matrigel were harvested by trypsinization or by using BD™ cell recovery solution, respectively. Total RNA was isolated from the harvested cells using RNeasy mini kit (Qiagen, Germany). First-strand cDNAs were synthesized with Omniscript reverse transcriptase kit (Qiagen) using random hexamer primers as per manufacturer's protocol. The relative expression of a STK35L1 transcript was measured by quantitative RT_PCR using PuReTaq Ready-To Go qPCR beads (GE Lifesciences) as per manufacturer's instructions. The data was normalized against the β-actin gene.

### Cell cycle analysis of endothelial cells

Endothelial cells, transfected with STK35L1 siRNA or control siRNA, were grown to confluence. Twenty-four hours after transfection, the cells were trypsinized, split in a 1∶2 ratio, replated and cultivated in serum-free medium for 24 hours. The cells were then released into the normal cell cycle progression by changing the medium to endothelial growth medium containing 10% FCS. The cells were harvested 6, 12, and 24 hours after release from starvation. Cells were fixed by adding 90% methanol drop-wise to the cell pellet. The cell suspension was kept for 30 minutes at 4°C and then cells were pelleted at 800 rpm followed by two times washing with PBS. Finally the cell pellet was resuspended in Propidium iodide, incubated at 37° for 1 hour and then analyzed by FACS (FACSCalibur flow cytometer, Becton Dickinson). The cell cycle data were analyzed by Modefit software.

### Cell-cycle RT^2^ Profiler™ PCR Array

Endothelial cells, transfected with STK35L1 siRNA or control siRNA were grown to confluence for 24 hours. The cells were trypsinized, splitted in a 1∶2 ratio, re-seeded and grown in endothelial cell basal medium (Promo cell) containing 0.5% serum for 24 hours. The cells were then released into normal cell cycle progression by changing the medium to complete endothelial cell growth medium containing 10% FCS. The cells were harvested six hours after release from starvation and total RNA was isolated by using the RNeasy mini kit (Qiagen). The RNA was reverse transcribed using the specific RT^2^ First Strand Kit (SA Biosciences). cDNA (2 µg) of STK35L1-silenced and control cells were mixed with RT^2^ qPCR Master Mix. The mixture was aliquoted into each well of the 96 well PCR array plate containing pre-dispensed gene specific primer sets. Quantitative real time PCR of 96 well plate was performed by using the iCycler (BioRad). Baseline and threshold values of real-time PCR were defined manually and were kept the same across the PCR array. The resulting threshold cycle values for all wells were exported to Microsoft Excel for use with the Data Analysis Template Excel file. For the analysis of these RT-PCR data, we used 4 control genes to calculate the normalization factor: β-2-microglobulin (B2M), hypoxanthine phosphoribosyl-transferase 1 (HPRT1), ribosomal protein L13a (RPL13A), and glyceraldehyde-3-phosphate dehydrogenase (GAPDH). Data were generated from three independent silencing experiments (n = 3). We considered the genes, whose expression was significantly up- or down-regulated by a factor of >4 (P value of ≤0.05).

### Matrigel cord formation assay

Matrigel® (BD Biosciences) was thawed on ice overnight, and 10 µl were pipetted with ice cold pipette tips into the lower chambers of an IBIDI angiogenesis slide (IBIDI GmbH,) and allowed to harden for 30 minutes at 37°C. After 48 hours of siRNA transfection, STK35L1-silenced and control cells were trypsinized and resuspended in complete endothelial growth medium. Transfected cells were more than 95% viable. The cell suspension (50 µl; 3×10^5^ cells/ml) was seeded on Matrigel into the well. The cells were incubated at 37°C and cord formation was observed on Nikon TE2000E-PFS fluorescence microscope. After six hours of seeding, pictures were taken and the images were analyzed with NIS Elements software. Network formation was quantified by measuring the total cord length and compared between silenced and non-silenced cells.

### Endothelial cell Migration after mechanical injury

HUVECs were seeded onto collagen coated six-well plate and following 24 hours of starvation the cell layer was scratched once from one edge to the other edge of the well using a pipette tip. The cells were washed with endothelial growth medium to remove cell debris and then incubated in complete endothelial growth medium. Wound healing was determined by measuring the cell-free area reaming in the wound, which is inversely correlates with the ability of the HUVECs to migrate.

### Endothelial cell migration using the IBIDI culture insert

An IBIDI culture insert (IBIDI GmbH) consists of two reservoirs separated by a 500 µm thick wall. For the endothelial migration assay, a BIDI culture insert was placed into one well of the 24 well plate and slightly pressed on the top to ensure tight adhesion. An equal number of control and STK35L1-silenced endothelial cell (70 µl; 4×10^5^ cells/ml) were added into the two reservoirs of the same insert and incubated at 37°C/5% CO_2_. After 10 hours, the insert was gently removed creating a gap of ∼500 µm. The well was filled with complete endothelial growth and the migration was observed by live cell imaging using Nikon TE2000E-PFS microscope.

## Supporting Information

Figure S1 **Protein sequence alignment of mammalian STK35L1.** N-terminal region and kinase domain of STK35L1 are shaded in gray and yellow color respectively. The conserved bipartite NLS (boxed) is marked in red color. Stretches of arginine and lysine are colored in gold.
(PDF)

Figure S2 **Endothelial cell prolifiration using AlamarBlue®.** HUVECs were seeded (25000 cells/well) in 24 well plates and were grown for 24 hours, 48 hours and 72 hours. Before four hours of every time points, cells were incubated with AlamarBlue reagent as described in Materials and methods. The absorbance of control siRNA treated cells was considered as 100% and the proliferation was calculated as % of control.
(JPG)

Table S1 **Prediction of protein-binding motifs within STK35L1 using the ELM web server.** Predicted binding motifs within STK35L1 are shown. The consensus binding sequence for the given binding domains is labeled in red. LIG, binding for.
(PDF)

Table S2 **List of genes and their position on the RT-PCR array 96 well plate.**
(PDF)

Movie S1 **Live cell imaging of human endothelial cells transfected with EGFP-PDM.** EGFP-PDM containing the PDZ-binding motif (see text for details), distributes thought the cytoplasm and the nucleus. In migrating cells, it concentrates in membrane ruffles at the leading edge as indicated by white arrows. Pictures were taken every four minutes for 90 minutes. Movie was edited with QuickTime Pro and iMovie software from Apple Inc.
(MOV)

Movie S2 **Migration assay using the IBIDI insert.** Endothelial cells transfected with STK35L1 siRNA (left side) or control siRNA (right side) were seeded into different reservoirs of an IBIDI insert. After 8 hours the insert was removed and the closure of the gap was observed on Nikon TE2000E-PFS fluorescence microscope equipped with incubation camber (37°C) and CO_2_ supply. The microscope function was controlled by NIS elements software. Pictures were taken every 7 minutes for 15 hours. Movie was edited with QuickTime Pro and iMovie software from Apple Inc.
(MOV)
