# Supplementary material for: STK35L1 Associates with Nuclear Actin and Regulates Cell Cycle and Migration of Endothelial Cells
Source: PLoS One. 2011 Jan 20;6(1):e16249. doi: 10.1371/journal.pone.0016249 (PMC3024402; doi:10.1371/journal.pone.0016249)
Supplement: Figure S1 — Protein sequence alignment of mammalian STK35L1. N-terminal region and kinase domain of STK35L1 are shaded in gray and yellow color respectively. The conserved bipartite NLS (boxed) is marked in red color. Stretches of arginine and lysine are colored in gold. (PDF) [file pone.0016249.s001.pdf]

Figure S1

```
STK35L1-Human         MGHQESPLARAPAGGAAYVKRLCKGLSWREHVESHGSLGAQASPASAAAA----EGSATR 56
STK35L1-Chimpanzee    MGHQESPLARAPAGGAAYVKRLCKGLSWREHVESHGSLGAQASPASAAAA----EGSATR 56
STK35L1-Cow           MGHQEPPLARLRAGRAAYIKRLRKGLSWREHVESRGSPDAQLSPESTAAVTRAVAGAVAR 60
STK35L1-Mouse         MGHQESPLTRAAAGGAAYIKRLRKVLSWRELGDGHGNLEAEASPGSVAVITRAAPRRATR 60
STK35L1-Rat           MGHQESPLTKVAAGGAAYIKRLRKVLSWRELEDSHGNLEAEASPGSAAVISGAAPRPATR 60

STK35L1-Human         RARAATSRAARSRRQPGPGADHPQAGAPGGKRAARKWRCAGQVTIQGPAPPRPRAGRRDE 116
STK35L1-Chimpanzee    RARATTSRAARSRRQPGPGADHPQAGAPGGKRAARKWRCAGQVTIQGPAPPRPRAGRRDE 116
STK35L1-Cow           PTRAAASRAAPYPRQPCPGADHPQPGALGGKRAARKWKGAGQVTIQGPALPRPGAGRRDE 120
STK35L1-Mouse         SARLPASRPTRLCRQARLGTDHPPARAPRGNRFARKRNSAGQITIQGPAPPHLGARRRDE 120
STK35L1-Rat           PTRLPASRPTRLCRQPGLGTDHPPARAPRGNRSARKRNSAGQITIQGTAPPHLRARRRDE 120

STK35L1-Human         AGGARAAPLLLPPPPAAMETGK-DGARRGTQSPERKRRSPVPRAPSTKLRPAAAARAMDP 175
STK35L1-Chimpanzee    AGGARAAPLLLPLPPAAMETGK-DGARRGTQSPERKRRSPVPRAPSTKLRPAAAAQAMDP 175
STK35L1-Cow           AGGSRAAPLLLPPPPAAMETGEEDGARRGTQSPERKRRSPVPRALSAKLRPAAAAQAMDP 180
STK35L1-Mouse         ARGARAAPLLLPPPPAAMETGKENGARRGTKSPERKRRSPVQRVLCEKLRP--AAQAMDP 178
STK35L1-Rat           AGGARAAPLLLPPPPAAMETGKENGARRRTKSPERKRRSPVQRVLCEKLRP--VAEAKDP 178

STK35L1-Human         VAAEAPGEAFLARRRPEGGGG--SARPRYSLLAEIGRGSYGVVYEAVAGRSGARVAVKKI 233
STK35L1-Chimpanzee    VAAEAPGEAFLARRRPEGGGG--SARPRYSLLAEIGRGSYGVVYEAVAGRSGARVAVKKI 233
STK35L1-Cow           VAAEAPGEAYLARRRPEGGGG--SARPRYSLLAEIGRGSYGVVYEAVAGRSGARVAVKKI 238
STK35L1-Mouse         AGAEVPGEAFLARRRPDGGGGDVPARPRYSLLAEIGRGSYGVVYEAVAGRSGARVAVKKI 238
STK35L1-Rat           AMAEVPGEAFLARRRPDGGGGDVPARPRYSLLAEIGRGSYGVVYEAVAGRSGAKVAVKKI 238

STK35L1-Human         RCDAPENVELALAEFWALTSLKRRHQNVVQFEECVLQRNGLAQRMSHGNKSSQLYLRLVE 293
STK35L1-Chimpanzee    RCDAPENVELALAEFWALTSLKRRHQNVVQFEECVLQRNGLAQRMSHGNKSSQLYLRLVE 293
STK35L1-Cow           RCDAPENVELALAEFWALTSLKRRHQNVVQFEECVLQRNGLAQRMSHGNKSSQLYLRLVE 298
STK35L1-Mouse         RCDAPENVELALAEFWALTSLKRRHQNIVQFEECVLQRNGLAQRMSHGNKNSQLYLRLVE 298
STK35L1-Rat           RCDAPENVELALAEFWALTSLKRRHQNIVQFEECVLQRNGLAQRMSHGNKNSQLYLRLVE 298

STK35L1-Human         TSLKGERILGYAEEPCYLWFVMEFCEGGDLNQYVLSRRPDPATNKSFMLQLTSAIAFLHK 353
STK35L1-Chimpanzee    TSLKGERILGYAEEPCYLWFVMEFCEGGDLNQYVLSRRPDPATNKSFMLQLTSAIAFLHK 353
STK35L1-Cow           TSLKGERILGYAEEPCYLWFVMEFCEGGDLNQYVLSRRPDPATNKSFMLQLTSAIAFLHK 358
STK35L1-Mouse         TSLKGERILGYAEEPCYLWFVMEYCEGGDLNQYVLSRRPDPATNKSFMLQLTSAIAFLHK 358
STK35L1-Rat           TSLKGERILGYAEEPCYLWFVMEYCEGGDLNQYVLSRRPDPATNKSFMLQLTSAIAFLHK 358

STK35L1-Human         NHIVHRDLKPDNILITERSGTPILKVADFGLSKVCAGLAPRGKEGNQDNKNVNVNKYWLS 413
STK35L1-Chimpanzee    NHIVHRDLKPDNILITERSGTPILKVADFGLSKVCAGLAPRGKEGNQDNKNVNVNKYWLS 413
STK35L1-Cow           NHIVHRDLKPDNILITERSGTPILKVADFGLSKVCAGLAPRGKEGNPDNKNVNVNKYWLS 418
STK35L1-Mouse         NHIVHRDLKPDNILITERSGTPILKVADFGLSKVCAGLAPRGKEGNQDNKNVNVNKYWLS 418
STK35L1-Rat           NHIVHRDLKPDNILITERSGTPILKVADFGLSKVCAGLAPRGKEGNQDNKDVNVNKYWLS 418

STK35L1-Chimpanzee    SACGSDFYMAPEVWEGHYTAKADIFALGIIIWAMIERITFIDSETKKELLGTYIKQGTEI 473
STK35L1-Human         SACGSDFYMAPEVWEGHYTAKADIFALGIIIWAMIERITFIDSETKKELLGTYIKQGTEI 473
STK35L1-Cow           SACGSDFYMAPEVWEGHYTAKADIFALGIIIWAMIERITFIDSETKKELLGTYIKQGTEI 478
STK35L1-Mouse         SACGSDFYMAPEVWEGHYTAKADIFALGIIIWAMIERITFIDSETKKELLGTYIKQGTEI 478
STK35L1-Rat           SACGSDFYMAPEVWEGHYTAKADIFALGIIIWAMIERITFIDSETKKELLGTYIKQGTEI 478

STK35L1-Human         VPVGEALLENPKMELHIPQKRRTSMSEGIKQLLKDMLAANPQDRPDAFELETRMDQVTCAA 534
STK35L1-Chimpanzee    VPVGEALLENPKMELHIPQKRRTSMSEGIKQLLKDMLAANPQDRPDAFELETRMDQVTCAA 534
STK35L1-Cow           VPVGEALLENPKMELHIPQKRRTSMSEGIKQLLKDMLAANPQDRPDAFELETRMDQVTCAA 539
STK35L1-Mouse         VPVGEALLENPKMELHIPQKRRTSMSEGVKQLLKDMLAANPQDRPDAFELETRMDQVTCAA 539
STK35L1-Rat           VPVGEALLENPKMELHIPQKRRTSMSEGVKQLLKDMLAANPQDRPDAFELETRMDQVTCAA 539
```
