# Supplementary figures and images for: STK35L1 Associates with Nuclear Actin and Regulates Cell Cycle and Migration of Endothelial Cells
Source: PLoS One. 2011 Jan 20;6(1):e16249. doi: 10.1371/journal.pone.0016249 (PMC3024402; doi:10.1371/journal.pone.0016249)

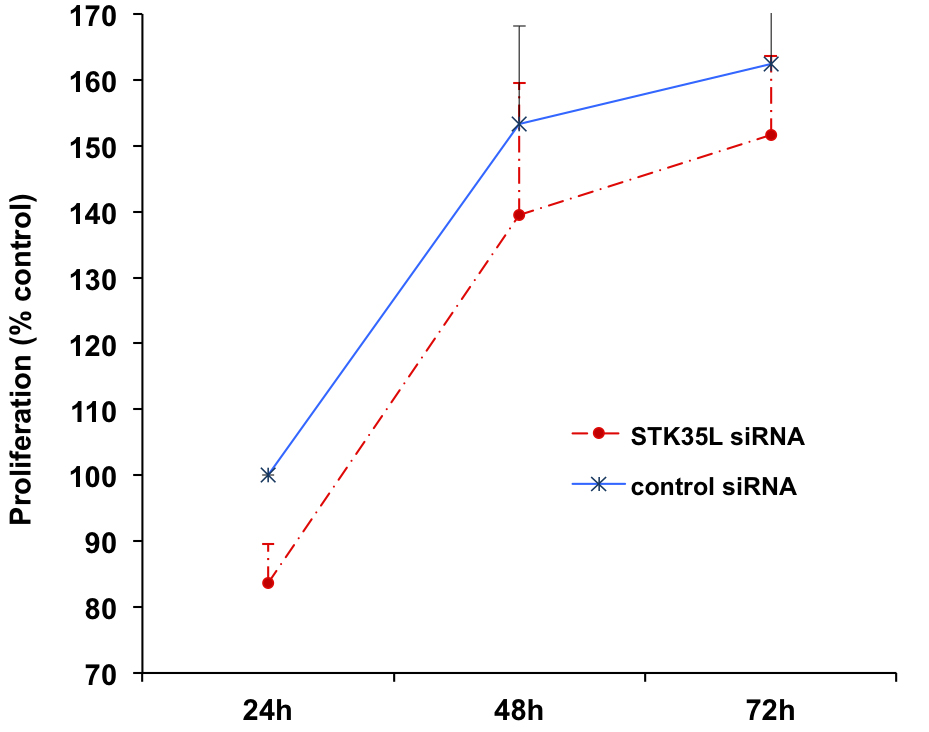

Supplement: Figure S2 — Endothelial cell prolifiration using AlamarBlue®. HUVECs were seeded (25000 cells/well) in 24 well plates and were grown for 24 hours, 48 hours and 72 hours. Before four hours of every time points, cells were incubated with AlamarBlue reagent as described in Materials and methods. The absorbance of control siRNA treated cells was considered as 100% and the proliferation was calculated as % of control. (JPG) [file pone.0016249.s002.jpg]
