# Supplementary material for: STK35L1 Associates with Nuclear Actin and Regulates Cell Cycle and Migration of Endothelial Cells
Source: PLoS One. 2011 Jan 20;6(1):e16249. doi: 10.1371/journal.pone.0016249 (PMC3024402; doi:10.1371/journal.pone.0016249)
Supplement: Table S1 — Prediction of protein-binding motifs within STK35L1 using the ELM web server. Predicted binding motifs within STK35L1 are shown. The consensus binding sequence for the given binding domains is labeled in red. LIG, binding for. (PDF) [file pone.0016249.s003.pdf]

Table S1 Prediction of protein-binding motifs within STK35L1 using the ELM web server.

Elm Name	Matched Sequence	Elm Description	Compartment	Pattern
LIG_FHA_2	134-METGKDG-140	Phosphothreonine motif binding a subset of FHA domains that have a preference for an acidic amino acid at the pT+3 position.	nucleus, Replication fork	..(T)..[DE].
LIG_MAPK_1	92-KWRCAGQVTI-101	MAPK interacting molecules (e.g. MAPKKs, substrates, phosphatases) carry docking motif that help to regulate spe-cific interaction in the MAPK cascade.	nucleus, cytosol	The classic motif approximates (R/K)xxxx#x# where # is a hydrophobic residue.
LIG_WW_4	3-HQESPL-8 40-AQASPA-45 144-GTQSPE-149 151-KRRSPV-156	Class IV WW domains interac-tion motif; phosphorylation-dependent interaction.	nucleus, cytosol	...[ST]P.
LIG_PDZ_3	173-MDPV176	Class III PDZ domains binding motif	cytosol, membrane,	.[DE].[IVL]
LIG_SH3_3	101-IQGPAPP-107 103-GPAPPRP-109 125-LLLPPPP-131 154-SPVPRAP160	This is the motif recognized by those SH3 domains with a non-canonical class I recognition specificity	cytosol, plasma mem-brane, focal adhesion	...[PV]..P
LIG_WW_3	105-APPRP109	WW domain of group III binding motif	cytosol	.PPR.
