# Supplementary material for: STK35L1 Associates with Nuclear Actin and Regulates Cell Cycle and Migration of Endothelial Cells
Source: PLoS One. 2011 Jan 20;6(1):e16249. doi: 10.1371/journal.pone.0016249 (PMC3024402; doi:10.1371/journal.pone.0016249)
Supplement: Table S2 — List of genes and their position on the RT-PCR array 96 well plate. (PDF) [file pone.0016249.s004.pdf]

Table S1: List of genes and their position on the RT-PCR array 96 well plate.

Position on plate	Unigene	Refseq	Symbol	Description	Gene name
A01	Hs.431048	NM_005157	ABL1	C-abl oncogene 1, receptor tyrosine kinase	ABL/JTK7
A02	Hs.533262	NM_013366	ANAPC2	Anaphase promoting complex subunit 2	APC2
A03	Hs.152173	NM_013367	ANAPC4	Anaphase promoting complex subunit 4	APC4
A04	Hs.194695	NM_004675	DIRAS3	DIRAS family, GTP-binding RAS-like 3	ARHI/NOEY2
A05	Hs.367437	NM_000051	ATM	Ataxia telangiectasia mutated	AT1/ATA
A06	Hs.271791	NM_001184	ATR	Ataxia telangiectasia and Rad3 related	FRP1/MEC1
A07	Hs.624291	NM_004324	BAX	BCL2-associated X protein	BCL2L4
A08	Hs.370292	NM_016567	BCCIP	BRCA2 and CDKN1A interacting protein	TOK-1/TOK1
A09	Hs.150749	NM_000633	BCL2	B-cell CLL/lymphoma 2	Bcl-2
A10	Hs.514527	NM_001168	BIRC5	Baculoviral IAP repeat-containing 5	API4/EPR-1
A11	Hs.194143	NM_007294	BRCA1	Breast cancer 1, early onset	BRCAI/BRCC1
A12	Hs.34012	NM_000059	BRCA2	Breast cancer 2, early onset	BRCC2/BROVCA2
B01	Hs.23960	NM_031966	CCNB1	Cyclin B1	CCNB
B02	Hs.194698	NM_004701	CCNB2	Cyclin B2	HsT17299
B03	Hs.430646	NM_005190	CCNC	Cyclin C	CycC
B04	Hs.523852	NM_053056	CCND1	Cyclin D1	BCL1/D11S287E
B05	Hs.376071	NM_001759	CCND2	Cyclin D2	KIAK0002
B06	Hs.244723	NM_001238	CCNE1	Cyclin E1	CCNE
B07	Hs.1973	NM_001761	CCNF	Cyclin F	FBX1/FBXO1
B08	Hs.79101	NM_004060	CCNG1	Cyclin G1	CCNG
B09	Hs.13291	NM_004354	CCNG2	Cyclin G2	Cyclin G2
B10	Hs.292524	NM_001239	CCNH	Cyclin H	CAK/p34
B11	Hs.279906	NM_001240	CCNT1	Cyclin T1	CCNT/CYCT1
B12	Hs.591241	NM_001241	CCNT2	Cyclin T2	FLJ90560
C01	Hs.374127	NM_003903	CDC16	Cell division cycle 16 homolog (S. cerevisiae)	APC6
C02	Hs.334562	NM_001786	CDC2	Cell division cycle 2, G1 to S and G2 to M	CDC28A/CDK1
C03	Hs.524947	NM_001255	CDC20	Cell division cycle 20 homolog (S. cerevisiae)	CDC20A/p55CDC
C04	Hs.514997	NM_004359	CDC34	Cell division cycle 34 homolog (S. cerevisiae)	E2-CDC34/UBC3

C05	Hs.19192	NM_001798	CDK2	Cyclin-dependent kinase 2	p33(CDK2)
C06	Hs.95577	NM_000075	CDK4	Cyclin-dependent kinase 4	CMM3/PSK-J3
C07	Hs.500015	NM_003885	CDK5R1	Cyclin-dependent kinase 5, regulatory subunit 1 (p35)	CDK5P35/CDK5R
C08	Hs.435952	NM_016408	CDK5RAP1	CDK5 regulatory subunit associated protein 1	C20orf34/C42
C09	Hs.119882	NM_001259	CDK6	Cyclin-dependent kinase 6	PLSTIRE
C10	Hs.184298	NM_001799	CDK7	Cyclin-dependent kinase 7	CAK1/CDKN7
C11	Hs.382306	NM_001260	CDK8	Cyclin-dependent kinase 8	K35
C12	Hs.370771	NM_000389	CDKN1A	Cyclin-dependent kinase inhibitor 1A (p21, Cip1)	CAP20/CDKN1
D01	Hs.238990	NM_004064	CDKN1B	Cyclin-dependent kinase inhibitor 1B (p27, Kip1)	CDKN4/KIP1
D02	Hs.512599	NM_000077	CDKN2A	Cyclin-dependent kinase inhibitor 2A (melanoma, p16, inhibits CDK4)	ARF/CDK4I
D03	Hs.72901	NM_004936	CDKN2B	Cyclin-dependent kinase inhibitor 2B (p15, inhibits CDK4)	CDK4I/INK4B
D04	Hs.84113	NM_005192	CDKN3	Cyclin-dependent kinase inhibitor 3	CDI1/CIP2
D05	Hs.24529	NM_001274	CHEK1	CHK1 checkpoint homolog (S. pombe)	CHK1
D06	Hs.291363	NM_007194	CHEK2	CHK2 checkpoint homolog (S. pombe)	CDS1/CHK2
D07	Hs.374378	NM_001826	CKS1B	CDC28 protein kinase regulatory subunit 1B	CKS1/PNAS-16
D08	Hs.83758	NM_001827	CKS2	CDC28 protein kinase regulatory subunit 2	CKSHS2
D09	Hs.146806	NM_003592	CUL1	Cullin 1	MGC149834
D10	Hs.82919	NM_003591	CUL2	Cullin 2	MGC131970
D11	Hs.372286	NM_003590	CUL3	Cullin 3	Cullin-Cul3
D12	Hs.443960	NM_004399	DDX11	DEAD/H (Asp-Glu-Ala-Asp/His) box polypeptide 11 (CHL1-like helicase homolog, S. cerevisiae)	CHL1/CHLR1
E01	Hs.211463	NM_004945	DNM2	Dynamin 2	CMTDI1/CMTDIB
E02	Hs.108371	NM_001950	E2F4	E2F transcription factor 4, p107/p130-binding	E2F-4
E03	Hs.80409	NM_001924	GADD45A	Growth arrest and DNA-damage-inducible, alpha	DDIT1/GADD45
E04	Hs.577202	NM_005316	GTF2H1	General transcription factor IIH, polypeptide 1, 62kDa	BTF2/TFB1
E05	Hs.386189	NM_016426	GTSE1	G-2 and S-phase expressed 1	B99
E06	Hs.26663	NM_016323	HERC5	Hect domain and RLD 5	CEB1/CEBP1
E07	Hs.152983	NM_004507	HUS1	HUS1 checkpoint homolog (S. pombe)	Hus1
E08	Hs.300559	NM_014708	KNTC1	Kinetochore associated 1	ROD
E09	Hs.594238	NM_002266	KPNA2	Karyopherin alpha 2 (RAG cohort 1, importin alpha 1)	IPOA1/QIP2

E10	Hs.591697	NM_002358	MAD2L1	MAD2 mitotic arrest deficient-like 1 (yeast)	HSMAD2/MAD2
E11	Hs.19400	NM_006341	MAD2L2	MAD2 mitotic arrest deficient-like 2 (yeast)	MAD2B/REV7
E12	Hs.477481	NM_004526	MCM2	Minichromosome maintenance complex component 2	BM28/CCNL1
F01	Hs.179565	NM_002388	MCM3	Minichromosome maintenance complex component 3	HCC5/P1-MCM3
F02	Hs.460184	NM_005914	MCM4	Minichromosome maintenance complex component 4	CDC21/CDC54
F03	Hs.517582	NM_006739	MCM5	Minichromosome maintenance complex component 5	CDC46/P1-CDC46
F04	Hs.689823	NM_002417	MKI67	Antigen identified by monoclonal antibody Ki-67	KIA
F05	Hs.509523	NM_002431	MNAT1	Menage a trois homolog 1, cyclin H assembly factor (Xenopus laevis)	MAT1/RNF66
F06	Hs.192649	NM_005590	MRE11A	MRE11 meiotic recombination 11 homolog A (S. cerevisiae)	ATLD/HNGS1
F07	Hs.492208	NM_002485	NBN	Nibrin	AT-V1/AT-V2
F08	Hs.147433	NM_182649	PCNA	Proliferating cell nuclear antigen	MGC8367
F09	Hs.531879	NM_002853	RAD1	RAD1 homolog (S. pombe)	HRAD1/REC1
F10	Hs.16184	NM_002873	RAD17	RAD17 homolog (S. pombe)	CCYC/HRAD17
F11	Hs.631709	NM_002875	RAD51	RAD51 homolog (RecA homolog, E. coli) (S. cerevisiae)	BRCC5/HRAD51
F12	Hs.655354	NM_004584	RAD9A	RAD9 homolog A (S. pombe)	RAD9
G01	Hs.408528	NM_000321	RB1	Retinoblastoma 1	OSRC/RB
G02	Hs.546282	NM_002894	RBBP8	Retinoblastoma binding protein 8	CTIP/RIM
G03	Hs.207745	NM_002895	RBL1	Retinoblastoma-like 1 (p107)	CP107/PRB1
G04	Hs.513609	NM_005611	RBL2	Retinoblastoma-like 2 (p130)	P130/Rb2
G05	Hs.487540	NM_002947	RPA3	Replication protein A3, 14kDa	REPA3
G06	Hs.269898	NM_013376	SERTAD1	SERTA domain containing 1	SEI1/TRIP-Br1
G07	Hs.23348	NM_005983	SKP2	S-phase kinase-associated protein 2 (p45)	FBL1/FBXL1
G08	Hs.81424	NM_003352	SUMO1	SMT3 suppressor of mif two 3 homolog 1 (S. cerevisiae)	DAP-1/GMP1
G09	Hs.79353	NM_007111	TFDP1	Transcription factor Dp-1	DP1/DRTF1
G10	Hs.379018	NM_006286	TFDP2	Transcription factor Dp-2 (E2F dimerization partner 2)	DP2/Dp-2
G11	Hs.654481	NM_000546	TP53	Tumor protein p53	LFS1/TRP53
G12	Hs.533273	NM_003334	UBA1	Ubiquitin-like modifier activating enzyme 1	A1S9/A1S9T
H01	Hs.534255	NM_004048	B2M	Beta-2-microglobulin	B2M
H02	Hs.412707	NM_000194	HPRT1	Hypoxanthine phosphoribosyltransferase 1	HGPRT/HPRT

H03	Hs.523185	NM_012423	RPL13A	Ribosomal protein L13a	RPL13A
H04	Hs.592355	NM_002046	GAPDH	Glyceraldehyde-3-phosphate dehydrogenase	G3PD/GAPD
H05	Hs.520640	NM_001101	ACTB	Actin, beta	PS1TP5BP1
H06	N/A	SA_00105	HGDC	Human Genomic DNA Contamination	HIGX1A
H07	N/A	SA_00104	RTC	Reverse Transcription Control	RTC
H08	N/A	SA_00104	RTC	Reverse Transcription Control	RTC
H09	N/A	SA_00104	RTC	Reverse Transcription Control	RTC
H10	N/A	SA_00103	PPC	Positive PCR Control	PPC
H11	N/A	SA_00103	PPC	Positive PCR Control	PPC
H12	N/A	SA_00103	PPC	Positive PCR Control	PPC
